# Strengthening Constitutive Motifs in Isotropic Fibrous Hydrogel for Exceptional Damage Resistance

**DOI:** 10.1002/advs.77852

**Published:** 2026-09-27

**Authors:** Bolin Wan, Xule Yang, Liju Xu, Dong Qiu

**Affiliations:** ^1^ Beijing National Laboratory for Molecular Sciences Laboratory of Polymer Physics and Chemistry CAS Research/Education Center for Excellence in Molecular Sciences Institute of Chemistry Chinese Academy of Sciences Beijing China; ^2^ School of Chemical Sciences University of Chinese Academy of Sciences Beijing China; ^3^ School of Chemistry and Chemical Engineering Northwestern Polytechnical University Xi'an China

**Keywords:** damage resistance, densification and crystallization, fibrous hydrogels, mechanical isotropy, polyvinyl alcohol

## Abstract

Super damage resistance requires high fracture energy in all directions, therefore, although fibrous hydrogels have demonstrated impressive reinforcing effects, their anisotropic nature usually prevents them from performing well. In this work, we propose a strategy to resolve this dilemma by strengthening constitutive motifs in isotropic fibrous hydrogel through nonsolvent quenching followed by salting‐out. This strategy generates isotropic nanofiber networks and subsequently reinforces those fibers via densification and crystallization, yielding dense and crystalline isotropic nanofiber (DCIF) networks that can efficiently transfer and dissipate stress to delay crack extension in all directions. Along any three orthogonal directions, the resultant DCIF network hydrogel demonstrates almost identically high strength of 20.15 ± 1.96 MPa, toughness of 126.48 ± 5.30 MJ/m^3^ and fracture energy of 242.01 ± 2.03 kJ/m^2^, achieving exceptional damage resistance in classical puncture and impact modes. This work provides a principle and methodology to fabricate and isotropically reinforce nanofiber hydrogels, thereby extending their applicability to versatile scenarios requiring isotropically mechanical superiority.

## Graphical abstract

An orientation‐free strategy is reported for fabricating damage‐resistant hydrogels by strengthening constitutive motifs in isotropic fibrous (IF) networks. Through nonsolvent quenching followed by salting‐out, IF networks are generated and their multiscale constituents are subsequently reinforced, yielding dense and crystalline isotropic nanofiber (DCIF) network hydrogels that hold potential applications requiring isotropic mechanical superiority and sustained damage resistance.

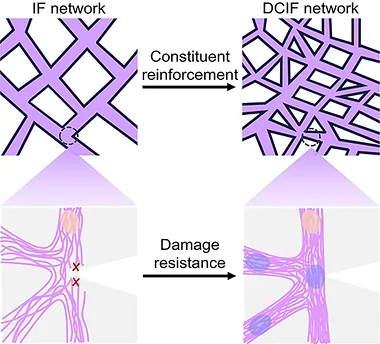

## 1 Introduction

Owing to high water content, synthetic hydrogels exhibit distinctive permeability while maintaining solid‐like behavior, having attracted extensive attention in cutting‐edge applications such as intelligent sensors and biomedical devices [1, 2, 3, 4, 5]. However, such high water content (i.e., low solid content) leads to low cohesive energy density and thereby inferior crack resistance, severely impairing their long‐term reliability [6, 7, 8]. In contrast, biological hydrogels such as tendon, ligament and cartilage show remarkable damage tolerance, arising from their robust collagen fiber networks [9, 10]. Enlightened by these natural tissues, incorporating exogenous fibers (e.g., aramid [11, 12], cellulose [13] and polydimethylsiloxane [14] fibers) into hydrogels has partially improved damage resistance. Nevertheless, the improvement is moderate due to the interfacial debonding caused by incompatibility between fibers and hydrogel matrices [15]. Alternatively, in‐situ fibrillation of polymer chains produces fibrous hydrogels that avoid interfacial incompatibility and significantly enhance the crack resistance. However, in‐situ fibrillation highly depends on structure orientation, such as mechanical training [16, 17, 18], directional freezing [19, 20] and stretch‐assisted confined drying [21]. Consequently, the resultant fibrous hydrogels demonstrate apparently anisotropic mechanical behavior, where the crack resistance along the alignment direction is improved while that in the perpendicular direction not, or even deteriorated [20]. Such mechanical anisotropy inevitably compromises their damage tolerance under complex and multiaxial mechanical loading [22], such as puncture and impact, where cracks may be initiated from any directions.

To improve comprehensive crack resistance for diverse applications, isotropically reinforced fibrous hydrogels are highly desirable. Accordingly, various strengthening strategies have been explored to improve isotropy [23, 24], including multidirectional alignment and oriented fiber assembly. For instance, bidirectional freeze‐casting is employed to fabricate fibrous hydrogels with aligned lamellar micro/nanostructures, which improves crack resistance along two orthogonal directions while leaving other directions unenhanced [25, 26]. Alternatively, helically stacking oriented fiber films into fibrous hydrogels, e.g., Bouligand‐type nanofibrous hydrogels, imparts two‐dimensional isotropy yet retains three‐dimensional anisotropy, thus constraining their utility in multiaxial mechanical loading [27, 28, 29]. By contrast, random spinning of polymer solutions produces three‐dimensionally isotropic fibrous hydrogels [30, 31]. However, the damage tolerance of them remains unsatisfactory even after interfacial welding, due to the insufficiency of fiber strength and interconnections [27, 32]. Overall, achieving exceptional damage resistance while preserving mechanical isotropy remains a formidable challenge in fibrous hydrogels.

The crack resistance and isotropy of fibrous hydrogels largely depend on the intrinsic robustness, interfacial connectivity and spatial orientation of their constituent fibers. Ideally, strengthening constituent fibers in isotropic fibrous hydrogels holds promise for achieving exceptional damage tolerance while maintaining mechanical isotropy [33]. In previous work, we have developed isotropic fibrous hydrogels via nonsolvent quenching, where poor solvency drives the aggregation and assembly of polymer chains into nanofibers [34]. Although the resultant isotropic fibrous hydrogels realized enhanced crack resistance compared to molecular hydrogels with identical compositions, they were still inferior to anisotropic hydrogels along the reinforced direction. Therefore, stronger driving forces to bolster polymer chain aggregation, such as salting‐out [35, 36, 37] and thermal annealing [38, 39, 40], are expected to further reinforce the isotropic fiber network constituents for superior isotropic damage resistance, which yet remains largely unexplored.

In this work, we developed a strategy to strengthen constitutive motifs within isotropic fibrous hydrogel by synergistically nonsolvent quenching and salting‐out, in order to achieve exceptional isotropic damage resistance. Specifically, nonsolvent quenching facilitated in‐situ formation of isotropic nanofiber (IF) networks, while subsequent salting‐out induced densification and crystallization of the IF network constituents, yielding highly dense and crystalline isotropic nanofiber (DCIF) networks. Without orientation, the DCIF networks can efficiently transfer and dissipate stress along all directions to prevent crack propagation. The resultant DCIF hydrogel realized identically high strength of 20.15 ± 1.96 MPa and toughness of 126.48 ± 5.30 MJ/m^3^ along any three orthogonal directions. Even when pre‐notched, the DCIF hydrogel maintained high strength of 3.25 ± 0.12 MPa and strain of 4.60 ± 0.20, resulting in ultrahigh fracture energy of 242.01 ± 2.03 kJ/m^2^. Such remarkable isotropic mechanical robustness endowed the DCIF hydrogel with excellent puncture and impact resistance. This work thus presented a universal strategy to regulate the formation and reinforcement of isotropic nanofibrous networks for isotropically damage‐tolerant hydrogels, paving a way toward application scenarios requiring sustained mechanical performance.

## 2 Results and Discussion

### 2.1 Design and Fabrication of the DCIF Network Hydrogel with Crack Resistance

**FIGURE 1:**
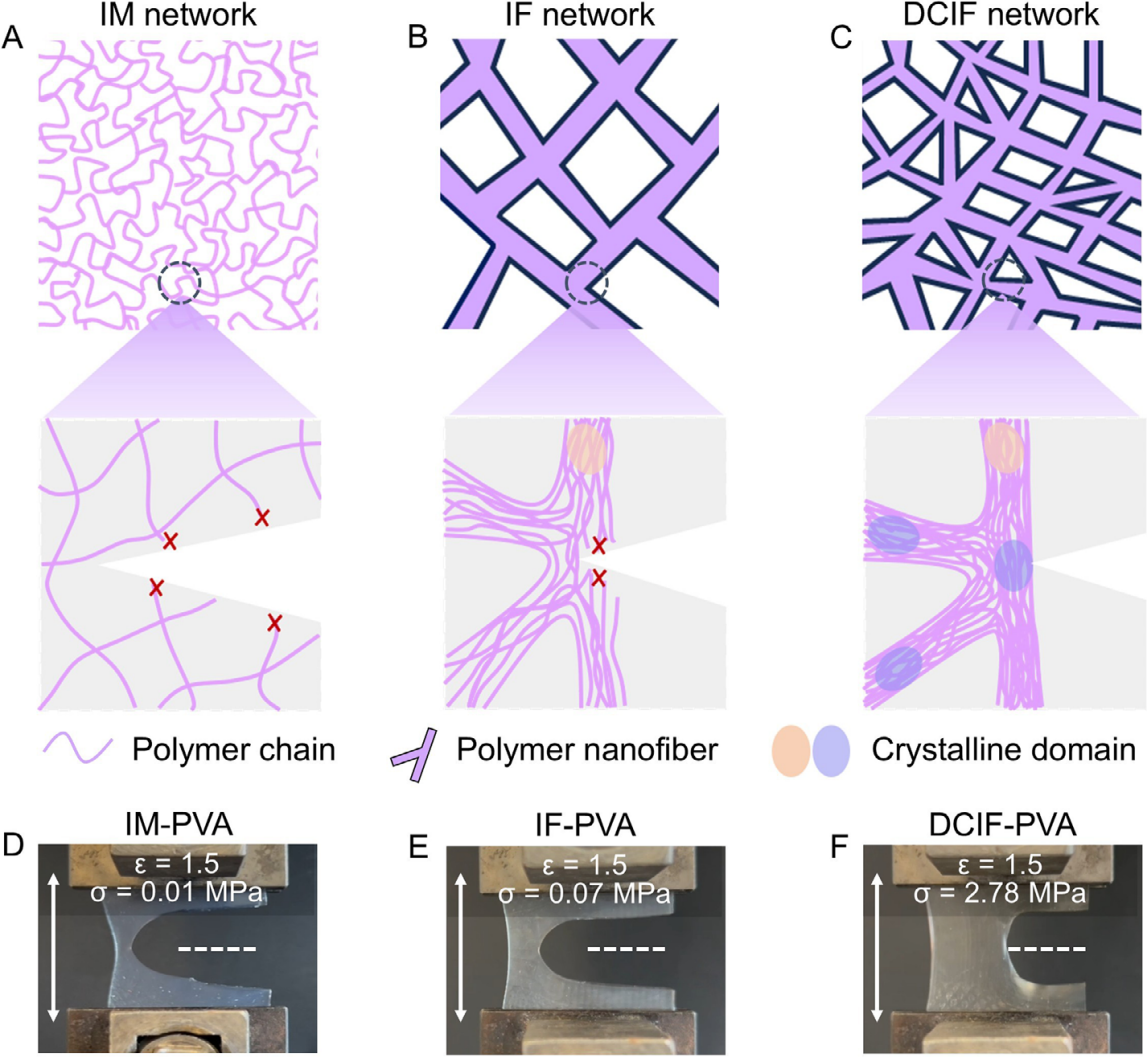
Schematic structure features and crack resistance mechanism of the IM, IF and DCIF network hydrogels. (A) The conventional IM network hydrogel was constructed with individual polymer chains that was too weak to hinder crack propagation. (B) Through nonsolvent quenching facilitated in‐situ fibrillation, the IF network hydrogel consisted of isotropic nanofibers (i.e., polymer chain bundles) with few crystalline domains, showing improved but still insufficient crack resistance. (C) Upon salting‐out to strengthen the IF network constituents, the DCIF network hydrogel was featured by denser nanofibers reinforced with more crystalline domains, which greatly reinforced the crack tip and efficiently arrested crack propagation. The optical photos of the pre‐notched (D) IM‐PVA, (E) IF‐PVA and (F) DCIF‐PVA hydrogels subjected to the same tensile strain of 1.5, showing that the notch grew slowly with crack pinning and blunting in the DCIF‐PVA hydrogel, while advancing quickly throughout the IF‐PVA and IM‐PVA hydrogels. The white arrows and dotted lines in (D–F) indicated the stretching directions and 2‐cm pre‐notches, respectively.

The network design of the DCIF hydrogel was illustrated in Figure 1, which was distinct from conventional isotropic molecular (IM) network and IF network hydrogels. Compared with the IM hydrogel based on the network of individual polymer chains that was too weak to hinder crack propagation (Figure 1A,D), the IF hydrogel consisting of isotropic nanofibers was expected to be mechanically stronger and tougher, because the nanofibers were more robust than single polymer chains (Figure 1B) [41]. Nevertheless, the crack resistance of the IF hydrogel remained insufficient owing to weak fibers with few crystalline domains (Figure 1E). Through densification and crystallization to reinforce constitutive motifs in the IF network, the enhanced DCIF hydrogel was developed, featuring denser while non‐orientated nanofibers with more crystalline domains (Figure 1C), which can efficiently transfer and dissipate stress along all directions to greatly reinforce the crack tip and delay crack extension (Figure 1F).

Following the above design, the DCIF‐polyvinyl alcohol (PVA) hydrogel was fabricated by an orientation‐free method, involving nonsolvent quenching and salting‐out (Figure S1). First, the IF‐PVA hydrogel was obtained through quenching a PVA/N‐methylpyrrolidone (NMP) gel in ethanol, followed by water swelling to induce an isotropic and loose nanofiber network. Upon salting‐out in sodium phytate aqueous solution to strengthen the IF network constituents (e.g., PVA chains and fibers) via densification and crystallization, the IF‐PVA hydrogel was converted into the DCIF‐PVA hydrogel. As another control, the IM‐PVA hydrogel was prepared by directly swelling the identical PVA/NMP precursor in water. Given that all three hydrogels shared identical chemical compositions, it enabled a head‐to‐head comparison to reveal how network constituents influence mechanical properties.

### 2.2 Structural Characteristics of the DCIF Network Hydrogel

**FIGURE 2:**
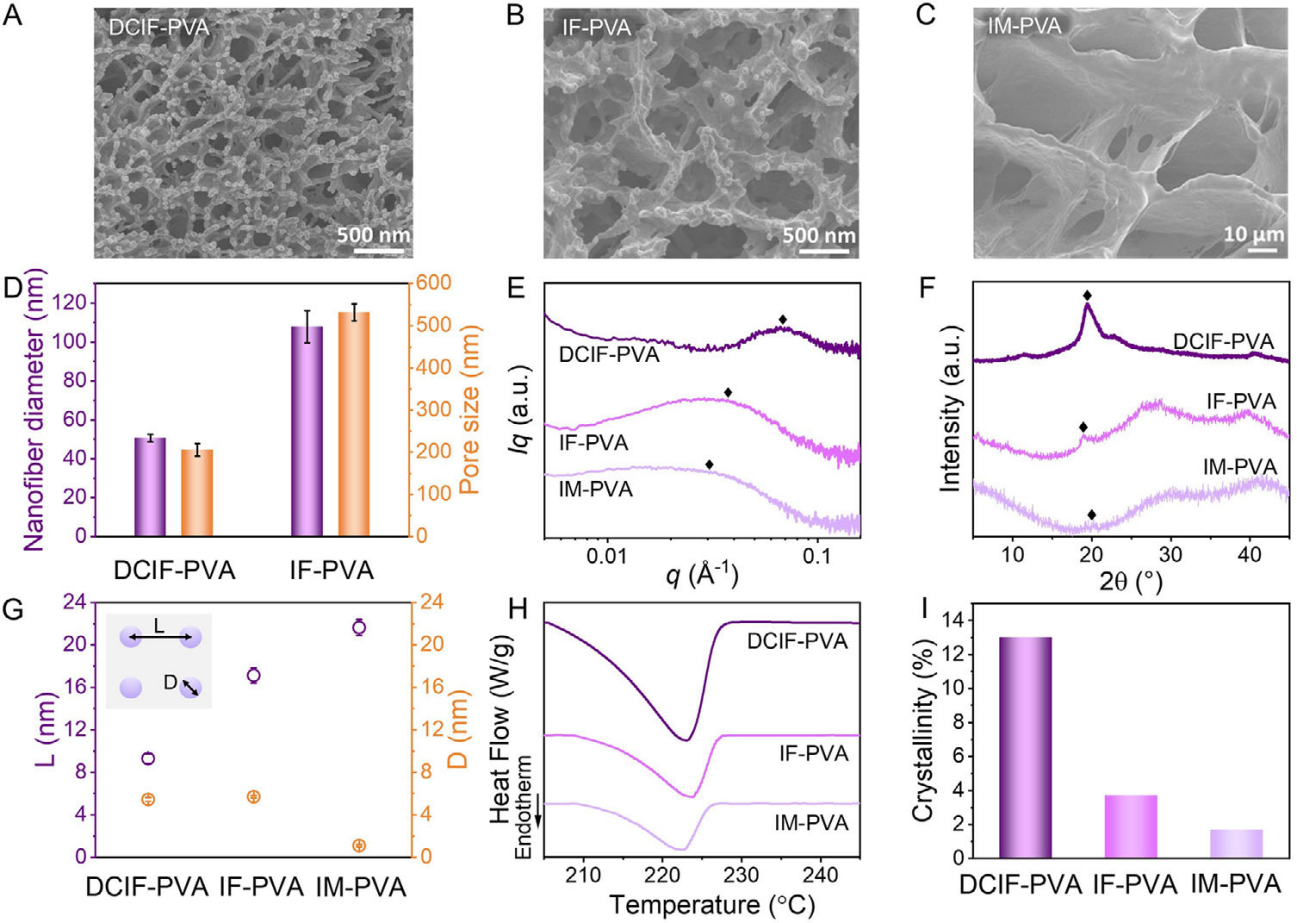
The structural characteristics of hydrogels. SEM images of (A) DCIF‐PVA, (B) IF‐PVA and (C) IM‐PVA hydrogels. The DCIF‑PVA hydrogel exhibited a dense nanofibrous network, whereas the IF‑PVA and IM‐PVA hydrogels showed a loose nanofibrous network and a sheet‐like porous network, respectively. (D) Statistic summary of nanofiber diameters and pore sizes of DCIF‐PVA and IF‐PVA hydrogels. The error bars represented the standard deviation; sample size n = 20. (E) SAXS profiles, (F) WAXS profiles and (G) the corresponding estimated average distance between adjacent crystalline domains (L) and average size of crystalline domains (D) of DCIF‐PVA, IF‐PVA and IM‐PVA hydrogels. The error bars represented the standard deviation; sample size n = 3. (H) DSC thermographs and (I) the corresponding crystallinities of DCIF‐PVA, IF‐PVA and IM‐PVA hydrogels.

To elucidate the reinforcing mechanisms, we investigated and compared the DCIF network structures with the IF and IM counterparts across multiple length scales. As observed in scanning electron microscopy (SEM) images at the micrometer scale, the DCIF‐PVA hydrogel displayed a dense nanofibrous network, whereas the IF‑PVA and IM‐PVA hydrogels showed a loose nanofibrous network and a sheet‐like porous network, respectively (Figure 2A–C). Quantitatively, the nanofiber diameter and pore size of the DCIF‐PVA hydrogel were 50.6 ± 1.9 nm and 205.7 ± 14.6 nm, much smaller than those (107.8 ± 8.3 nm and 532.0 ± 19.8 nm) of the IF‐PVA hydrogel (Figure 2D). These observations suggested that nonsolvent quenching facilitated in‐situ formation of IF networks, whose constituent nanofibers were subsequently densified and reinforced by salting‐out.

Furthermore, small‐angle X‐ray scattering (SAXS) and wide‐angle X‐ray scattering (WAXS) were used to study the structural evolution of the DCIF network at the nanometer scale. SAXS measurements were performed on the hydrogel samples in the swollen state, and the scattering intensity was multiplied with the scattering vector (*q*) to clearly identify the position of the diffraction peak (Figure 2E). According to the Bragg equation (L = 2π/*q*_max_), the average distance between adjacent crystalline domains (L) was calculated from the *q* at the peak position (*q*_max_). Compared with the IM‐PVA hydrogel that showed a peak at a lower *q*_max_ of ≈0.030 Å^−1^ and corresponding larger L of ≈21 nm, the IF‐PVA hydrogel exhibited a peak at a higher *q*_max_ of ≈0.036 Å^−1^, related to a decreased L of ≈17 nm, indicating that the latter was denser than the former due to nonsolvent quenching. After salting‐out, the peak of the DCIF‐PVA hydrogel was shifted to a much higher *q*_max_ of ≈0.067 Å^−1^, corresponding to a narrower L of ≈9.3 nm, along with an additional shoulder peak emerging in the low‐*q* region, likely attributable to nanofiber aggregates, collectively confirming further densification and structural reinforcement of the IF networks [34, 37].

Additionally, WAXS measurements were also carried out on the hydrogel samples in the swollen state. As shown in Figure 2F, the DCIF‐PVA hydrogel showed a substantially stronger diffraction peak at 2θ of 19.7° than the IF‐PVA hydrogel, whereas the IM‐PVA hydrogel had no apparent diffraction peak [37]. Moreover, other two small peaks at 2θ of 11.6°and 23.1° became evident in the DCIF‐PVA hydrogel, manifesting the salting‐out fostered crystallization. Quantitatively, the average size of crystalline domains (D) can be estimated using the Scherrer's equation [D = kλ/(βcosθ)]. As compared in Figure 2G, D markedly increased from ∼1.1 nm for the IM‐PVA hydrogel to ≈5.7 nm for the IF‐PVA hydrogel, confirming the substantial crystallization driven by nonsolvent quenching. Strikingly, D (≈5.5 nm) of the DCIF‐PVA hydrogel remained almost the same as IF‐PVA hydrogel, probably due to the strong restriction of PVA segments in the IF‐PVA network. Therefore, the generation of new crystalline domains was favored over further growth of existing ones during salting‐out [42], which was believed to account for the DCIF‐PVA networks being cross‐linked by a greater number of crystalline domains.

To further quantify the evolution of crystalline morphology, we measured the melting temperature and crystallinity of the hydrogel samples in the swollen state by differential scanning calorimetry (DSC). In comparison to the IM‑PVA hydrogel, the melting peak intensity of the DCIF‑PVA hydrogel increased as well as the melting temperature (Figure 2H,I), indicating the formation of more compact crystalline domains. Quantitatively, the crystallinity of the DCIF‐PVA hydrogel was significantly improved to 13.0% from 3.7% and 1.6% of the IF‐PVA and IM‐PVA hydrogels, respectively. Given the influence of solid content, we normalized the crystallinity by the solid content. The normalized crystallinity of the DCIF‑PVA hydrogel was 24 %, still higher than those of the IF‑PVA (12 %) and IM‑PVA (13 %) hydrogels. It is worth noting that the melting temperature in DCIF‐PVA hydrogel was close to IF‐PVA hydrogel, suggesting that the increased crystallinity of the former was indeed due to the formation of more crystallites rather than crystallite growth, in agreement with the WAXS observations. Overall, the nonsolvent quenching combined with salting‐out strengthened multiscale structures in the IF‐PVA hydrogel via densification and crystallization, endowing the DCIF‐PVA hydrogel with isotropic and remarkable crack resistance.

### 2.3 Exceptional Isotropic Crack Resistance of the DCIF Network Hydrogel

**FIGURE 3:**
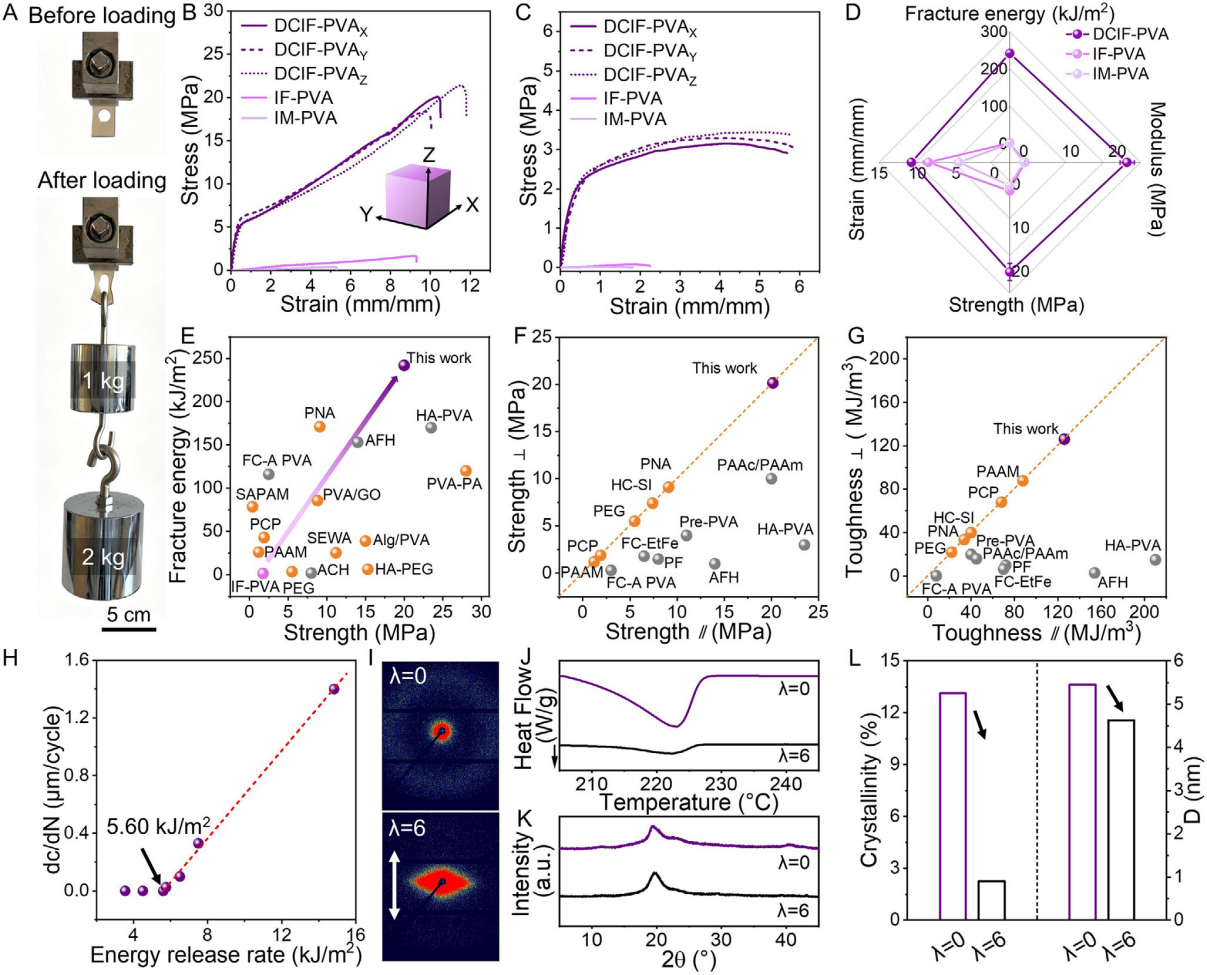
Mechanical properties of the DCIF‐PVA network hydrogel and its structural evolution during deformation. (A) The photographs of a perforated DCIF‐PVA hydrogel with a cross‐sectional area of 16.2 mm^2^ and a hole of 78.5 mm^2^ lifting a 3 kg weight without further hole cracking, indicating its ultrahigh damage tolerance under heavy loads. Representative tensile stress‐strain curves of (B) unnotched and (C) notched DCIF‐PVA hydrogels along three orthogonal directions (denoted as X, Y, Z directions), as well as IF‐PVA and IM‐PVA hydrogels. (D) The radar map comparing comprehensive mechanical properties of DCIF‐PVA, IF‐PVA and IM‐PVA hydrogels, including fracture energy, modulus, strength and strain. The error bars represented the standard deviations; sample size n = 3. (E) The mechanical performances of DCIF‐PVA hydrogel in the fracture energy‐strength diagram, together with results from previously reported high‐performance hydrogels, demonstrating that the DCIF‐PVA hydrogel possessed excellent strength and crack‐resistance simultaneously. Comparison chart by plotting (F) strength and (G) toughness in the parallel direction versus perpendicular direction among the developed DCIF‐PVA hydrogel, other reported isotropic and anisotropic hydrogels. The orange and gray dots represented isotropic and anisotropic hydrogels, respectively. The data used were summarized in Table S1. (H) Fatigue resistance of the DCIF‐PVA hydrogel. (I) 2D SAXS patterns of DCIF‐PVA hydrogel at varied tensile strains (λ = 0 and 6). (J) DSC thermograms, (K) WAXS profiles, and (L) the corresponding crystalline domain sizes and crystallinities of DCIF‐PVA hydrogel under varied tensile strains (λ = 0 and 6). The error bars represented the standard deviation; sample size n = 3. The white arrow in I and black arrow in J indicated the stretching direction and endothermic direction, respectively.

Despite sharing identical chemical compositions, DCIF‐PVA, IF‐PVA and IM‐PVA hydrogels demonstrated substantially different mechanical performances owing to their distinct network structures. With the elaborate DCIF network to efficiently transfer and dissipate stress, the DCIF‐PVA hydrogel was isotropically strong and damage‐tolerant. As proof in Figure 3A, a perforated DCIF‐PVA hydrogel strip with a cross‐sectional area of 16.2 mm^2^ and a hole of 78.5 mm^2^ can lift a 3 kg weight without noticeable deformation and further hole cracking. Quantitatively, the tensile strength of the DCIF‐PVA hydrogel reached 20.15 ± 1.96 MPa (Figure 3B), approximately 11‐fold and 50‐fold as high as those of the IF‐PVA (1.75 ± 0.11 MPa) and IM‐PVA (0.43 ± 0.04 MPa) hydrogels, respectively. Notably, even under pre‐notched conditions, the DCIF‐PVA hydrogel still displayed the highest critical strength of 3.25 ± 0.12 MPa, one to two orders of magnitude higher than the IF‐PVA and IM‐PVA hydrogels (Figure 3C). Also, the notched DCIF‐PVA hydrogel exhibited the largest critical strain of 4.60 ± 0.20, compared with the IF‐PVA (1.80 ± 0.11) and IM‐PVA (1.30 ± 0.10) counterparts (Figure S2), indicating the efficient suppression of crack propagation. Consequently, a significant improvement in the fracture energy was achieved, i.e., from 0.07 ± 0.01 kJ/m^2^ for the IM‐PVA hydrogel and 1.76 ± 0.12 kJ/m^2^ for the IF‐PVA hydrogel to 242.01 ± 2.03 kJ/m^2^ for the DCIF‐PVA hydrogel. Nevertheless, the water content of the DCIF‐PVA hydrogel (44.87 ± 1.05%) was lower than those of IF‐PVA (69.50 ± 0.71%) and IM‐PVA (88.01 ± 1.41%) hydrogels, which also had effect on the mechanical properties (Figure S3). To rule out this effect, we prepared IF‐PVA and IM‐PVA hydrogels with comparable water content (∼40%) to the DCIF hydrogel and compared their mechanical properties. As shown in Figure S4, even with the nearly identical water content, the tensile modulus and strength of IF‐PVA (0.58 ± 0.01 MPa and 2.95 ± 0.04 MPa) and IM‐PVA (0.09 ± 0.01 MPa and 1.41 ± 0.08 MPa) hydrogels were still much lower than those of the DCIF hydrogel (22.10 ± 1.52 MPa and 20.15 ± 1.96 MPa), underscoring the contribution of the DCIF network to the enhanced mechanical performance. Additionally, it was noteworthy that the Sal‐IM‐PVA hydrogel fabricated by salting‐out the conventional IM‐PVA hydrogel showed much lower modulus (3.71 ± 0.17 MPa), strength (12.45 ± 1.16 MPa) and fracture energy (65.89 ± 1.79 kJ/m^2^) even at a higher solid content (59.60 ± 0.82%) and crystallinity (16.55%), further highlighting the key role of the DCIF network for enhanced mechanical properties (Figures S5–S7). Overall, the developed DCIF‐PVA hydrogel demonstrated extraordinary comprehensive mechanical properties, outperforming most existing hydrogels (Figure 3D,E) [13, 20, 26, 37, 43, 44, 45, 46, 47, 48, 49, 50, 51, 52].

Unlike most reported nanofibrous hydrogels with anisotropic mechanical behavior [43], our developed DCIF‐PVA hydrogel exhibited equally enhanced mechanical properties in all directions due to the lack of orientation. For both unnotched and notched DCIF‐PVA hydrogels, the tensile behavior was almost identical along any three orthogonal directions (Figure 3B,C and Figure S8). As summarized and compared in Figure 3F,G, the DCIF‐PVA hydrogel simultaneously achieved identically high strength (20.15 ± 1.96 MPa) and toughness (126.48 ± 5.30 MJ/m^3^) along any two orthogonal directions, far exceeding most isotropic hydrogels and even rivaling most anisotropic hydrogels in their reinforced directions [20, 27, 43, 44, 45, 46, 47, 52, 53, 54, 55, 56]. Moreover, owing to its isotropic and homogeneous network architecture, the developed DCIF hydrogel also demonstrated superior stiffness and toughness under compression, thereby broadening its potential utilities. As shown in Figure S9, the DCIF hydrogel achieved both high compressive modulus of 6.45 ± 0.07 MPa and compressive toughness of 7.52 ± 0.12 MJ/m^3^, exceeding those of IF‐PVA hydrogel (0.47 ± 0.05 MPa and 1.45 ± 0.54 MJ/m^3^) by 14 and 5 times, respectively. These results further highlighted the critical role of the strengthened constitutive motifs within DCIF network for enhancing mechanical performance.

Furthermore, we evaluated the energy dissipation capability of the DCIF hydrogel through the cyclic tensile tests under varied strains. As shown in Figure S10, the DCIF hydrogel exhibited substantially larger hysteresis loops compared with IF‐PVA and IM‐PVA hydrogels, manifesting its superior energy dissipation performance. Quantitatively, when the tensile strain increased from 1 to 9, the corresponding dissipated energy and dissipation coefficient of the DCIF hydrogel significantly improved from 2.44 ± 0.26 MJ/m^3^ and 62.59 ± 3.43% to 35.88 ± 2.10 MJ/m^3^ and 83.37 ± 1.85%, much higher than those of IF‐PVA (3.09 ± 0.40 MJ/m^3^ and 67.85 ± 1.58% at strain of 7) and IM‐PVA (0.03 ± 0.01 MJ/m^3^ and 23.53 ± 3.71% at strain of 3) hydrogels at the maximum pre‐fracture strain, providing efficient energy dissipation to toughen the DCIF hydrogel. Also, fatigue resistance is another critical indicator of damage tolerance for applications involving long‐term dynamic loads. Notably, the DCIF‐PVA hydrogel exhibited a high fatigue threshold of 5.60 kJ/m^2^ (Figure 3H). To verify this value, a notched sample was subjected to 5000 loading‐unloading cycles at an energy release rate of 5.54 kJ/m^2^, and no crack propagation was observed, underscoring its excellent fatigue resistance (Figure S11).

These reinforced constitutive motifs in the DCIF network, such as nanofibers and crystalline domains, were not rigid, but rather adaptive during deformation. At the tensile strain of 0, the 2D SAXS diffraction pattern was isotropic, and the corresponding azimuthal integration curve was nearly flat, indicating the isotropic network structure. As the strain increased to 6, the 2D SAXS diffraction patterns displayed more pronounced peaks perpendicular to the stretching direction, and the corresponding azimuthal integration curve exhibited a peak at the azimuthal angle of 90°, suggesting the strain induced nanofiber alignment (Figure 3I and Figure S12). Additionally, both crystallinity and crystalline domain size decreased (Figure 3J–L), indicating the crystalline domain dissociation. Notably, the intensity of the melting peak decreased as well as the melting temperature, owing to the damage and looseness of crystalline domains. Collectively, the DCIF network can efficiently transfer and dissipate stress along all directions via nanofiber orientation and crystalline domain dissociation, which was key to the achievement of isotropic and outstanding damage resistance.

Based on the strengthening mechanism of DCIF‐PVA hydrogels, we propose that reinforcing the constitutive motifs in isotropic nanofibrous networks is a universal strategy to isotropically strengthen and toughen hydrogels, thereby improving their damage resistance. To validate this hypothesis, we employed other kosmotropic salts as the salting‐out medium, such as sodium citrate (CA). As expected, the DCIF‐PVA‐CA hydrogel demonstrated a superior tensile modulus of 7.56 ± 0.52 MPa and tensile strength of 11.08 ± 0.91 MPa, outperforming the Sal‐IM‐PVA‐CA hydrogel with the modulus of 4.93 ± 0.23 MPa and strength of 4.69 ± 0.54 MPa (Figure S13). Even when pre‐notched, the DCIF‐PVA‐CA hydrogel also exhibited a higher fracture energy (112.16 ± 2.02 kJ/m^2^) than that (53.93 ± 1.36 kJ/m^2^) of the Sal‐IM‐PVA‐CA hydrogel, manifesting its superior crack resistance (Figure S14). Moreover, thermal annealing is capable of boosting crystallization and densification of hydrogel networks [57]. Without surprise, the tensile modulus, strength and fracture energy of the DCIF‐PVA‐annealing hydrogel was 1.61 ± 0.20 MPa, 4.43 ± 0.80 MPa and 0.65 ± 0.05 kJ/m^2^, also much higher than those (tensile modulus of 0.18 ± 0.11 MPa, tensile strength of 1.37 ± 0.15 MPa and fracture energy of 0.25 ± 0.02 kJ/m^2^) of the IM‐PVA‐annealing hydrogel (Figures S15 and S16). Collectively, if one could find other suitable methods to strengthen constituent motifs in isotropic fiber networks, it is possible to develop damage tolerant hydrogels for broader applications either.

### 2.4 Superior Puncture and Impact Resistance of the DCIF Network Hydrogel

**FIGURE 4:**
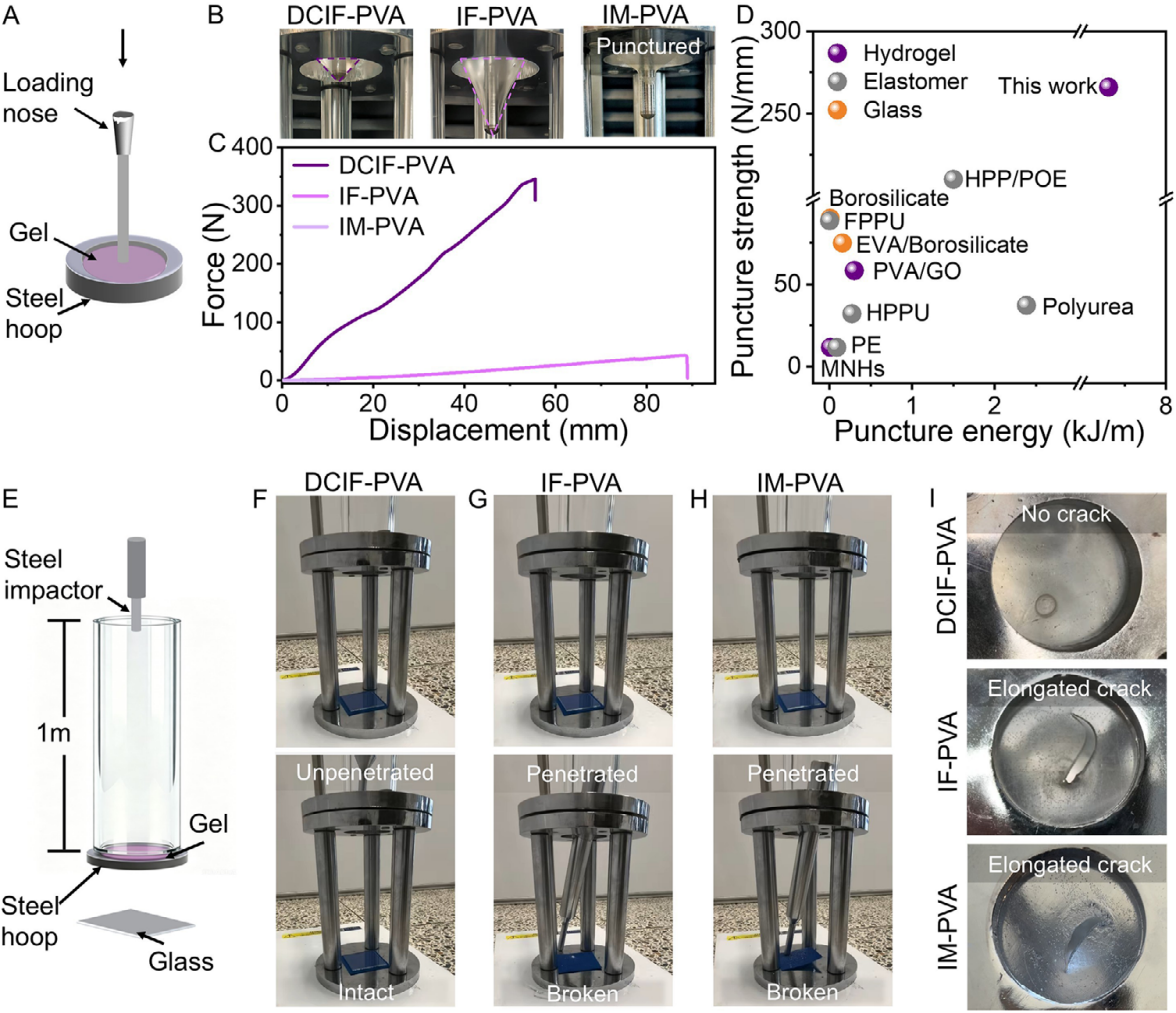
Superior puncture and impact resistance of the DCIF‐PVA hydrogel. (A) Schematic diagram of the puncture test setup wherein a loading nose was punctured onto the hydrogel sample until penetration. (B) Deformation comparison for DCIF‐PVA, IF‐PVA and IM‐PVA hydrogels under an identical puncture force of 40 N. (C) Representative plots of the puncture force against displacement for DCIF‐PVA, IF‐PVA and IM‐PVA hydrogels. (D) Comparison chart plotting the puncture strength versus puncture energy for different materials, including hydrogels, elastomers and glass. (E) Schematic illustration of the custom‐designed drop‐weight impact test apparatus. Photographs of (F) DCIF‐PVA, (G) IF‐PVA and (H) IM‐PVA hydrogels and the corresponding underlying glass slides before (top) and after (bottom) impacting by a 400 g steel impactor from the same height of 1 m. (I) Top‐view images of DCIF‐PVA, IF‐PVA and IM‐PVA hydrogels after impact. In striking contrast to the IF‐PVA and IM‐PVA hydrogels that failed to withstand the impact and thus left the underlying glass slides shattered, the DCIF‐PVA hydrogel well resisted the impact and protected the underlying glass slide from breaking, revealing its superior damage tolerance.

Given its omnidirectional crack resistance, the DCIF network hydrogel holds great promise for applications involving complex and multiaxial mechanical loading scenarios, such as puncture and impact tolerance where the crack propagation must be hindered in any direction. To evaluate its puncture resistance, the quasi‐static puncture test was carried out by gradually punching a stainless‐steel loading nose onto the hydrogel sample at a speed of 10 µm/s until perforation (Figure 4A). As displayed in Figure 4B, under the same puncture force of 40 N, the DCIF‐PVA hydrogel exhibited slighter deformation compared with the IF‐PVA hydrogel undergone large deformation, while the IM‐PVA hydrogel was easily punctured through. Quantitatively, the DCIF‐PVA hydrogel realized the maximum puncture strength of 271.03 ± 18.01 N/mm and a puncture energy of 7.70 ± 0.10 kJ/m (Figure 4C, Figure S17). Notably, in contrast to conventional rigid puncture‐resistant materials (e.g., glass) and soft ones (e.g., elastomers and hydrogels) that typically exhibited a trade‐off between puncture strength and puncture energy, the DCIF‐PVA hydrogel achieved a rare combination of those two (Figure 4D), highlighting its potential as a desirable puncture‐resistant protective material.

Moreover, the drop‐weight impact test was performed to demonstrate the impact resistance of the DCIF‐PVA hydrogel, where a 400 g stainless‐steel impactor (spherical tip radius: 4 mm) was dropped from a height of 1 m onto the hydrogel sheet to determine whether it would penetrate the hydrogel sheet and shatter the underlying glass slide (Figure 4E). As shown in Figure 4F–I and Movies S1 and S2, the IM‐PVA hydrogels ruptured easily upon impact, exhibiting a large elongated crack, and thus the underlying glass slide underwent explosive fragmentation. Similarly, the less damage‐tolerant IF‐PVA hydrogel failed to protect the underlying glass slides from breaking, also leaving an elongated crack. In sharp contrast, the DCIF‐PVA hydrogel effectively resisted the impact and kept the underlying glass slide intact, demonstrating its superior damage resistance. Overall, the isotropically fracture‐resistant DCIF‐PVA hydrogel demonstrated exceptional puncture and impact resistance, potentially applicable in scenarios like human motion, transient protection and transportation.

## 3 Conclusions

To conclude, we proposed that strengthening multiscale constituents in isotropic fibrous hydrogels could achieve exceptional damage resistance. By combining nonsolvent‐quenching and salting‐out, the DCIF‐PVA hydrogel was fabricated to validate this strategy. Owing to the omnidirectional stress transfer and dissipation across multiple scales via nanofiber alignment, sliding and pull‐out, together with crystalline domain dissociation, the DCIF‐PVA hydrogel exhibited identically high strength (20.15 ± 1.96 MPa), toughness (126.48 ± 5.30 MJ/m^3^) and fracture energy (242.01 ± 2.03 kJ/m^2^) along any three orthogonal directions, collectively conferring outstanding puncture and impact resistance. We anticipate that reinforcement of constitutive motifs in isotropic nanofibrous networks is a universal strategy to isotropically strengthen and toughen hydrogels, thereby broadening their applicability to complex load‐bearing environments, such as body armor, soft robotics and biomedical devices.

## 4 Experimental Section

### 4.1 Preparation of the DCIF‐PVA Hydrogel

The nonsolvent‐quenching combined with salting‐out method was adopted to fabricate the DCIF‐PVA hydrogel. Specifically, a PVA solution was first prepared by dissolving 6 g of PVA powder in 44 mL of NMP under constant stirring at 120 °C for 4 h. After centrifugal defoaming, the resulting clear PVA solution was cast into a pre‐designed Teflon mold and subjected to gelation at ‐25 °C for 12 h to form the PVA‐NMP gel. Meanwhile, a sodium phytate solution was formulated by diluting phytic acid solution with NaOH solution to the desired concentration (1 M) with pH = 7. Subsequently, the PVA‐NMP gel was sequentially immersed in ethanol, deionized water and sodium phytate solution, each for 24 h at room temperature, to fabricate the DCIF‐PVA hydrogel. For comparison, the IF‐PVA hydrogel was prepared by soaking the PVA‐NMP gel in ethanol for 24 h and then reswelling in deionized water for 24 h. The IM‐PVA hydrogel was fabricated by directly immersing the PVA‐NMP gel in deionized water for 24 h. The Sal‐IM‐PVA hydrogel was prepared by immersing the IM‐PVA hydrogel in a 1 M sodium phytate solution for 24 h.

Similarly, the DCIF‐PVA‐CA and Sal‐IM‐PVA‐CA hydrogels were prepared by soaking the IF‐PVA and IM‐PVA hydrogels in a 1.5 M sodium citrate solution, respectively. The DCIF‐PVA‐annealing and IM‐PVA‐annealing hydrogels were obtained by drying the IF‐PVA hydrogel and IM‐PVA hydrogel at room temperature for 6 h and then in a blast drying oven at 120 °C for 1 h, followed by reswelling in water for 48 h.

### 4.2 Scanning Electron Microscope (SEM)

The hydrogel samples were frozen and fractured in liquid nitrogen, and subsequently freeze‐dried using an LGJ‐30 freeze dryer to yield dried samples. Before SEM observation, the fractured surfaces of the freeze‐dried samples were sputter‐coated with a thin platinum layer. The microscopic morphology of the freeze‐dried samples was characterized using a SEM (JSM 7500F) at an acceleration voltage of 3 kV. Nano Measurer software was used to measure nanofiber diameters and pore sizes of DCIF‐PVA and IF‐PVA hydrogels (sample size n ≥20).

### 4.3 X‐Ray Scattering Tests

The rectangular hydrogel samples with a length of 30 mm, a width of 10 mm and a thickness of 1 mm were rinsed with deionized water to remove excess salt from the sample surfaces before testing. The small‐angle X‐ray scattering (SAXS) measurements were carried out at Xeuss 2.0 SAXS/WAXS System (Xenocs, France) with an X ray of λ = 0.154 nm. The sample‐to detector distance was 2500 mm, covering a momentum transfer (*q*) range of 0.003–0.185Å^−1^, and the exposure time was set as 900 s. According to the Bragg expression (L = 2π/q_max_), the average distance between adjacent crystalline domains (L) can be estimated from the *q* at the peak position (*q*_max_).

The wide‐angle X‐ray scattering (WAXS) measurements were carried out at the Rigaku (D/MAX 2500) instrument with an X ray of λ = 0.154 nm, operated at 40 kV and 200 mA, and the WAXS profiles were collected in the 2θ range of 5° to 50° with a speed at 2°/min. The average size of crystalline domains (D) can be estimated using the Scherrer's equation [D = kλ/(βcosθ)], where k was the dimensionless shape factor set as 1 for approximately spherical crystalline domain, λ was the wavelength of X‐ray diffraction, β was the width at half maximum of the peak, and θ was the Bragg angle.

### 4.4 Crystallinity Content Measurement

Differential scanning calorimeter (TA Instruments, DSC Q2000) was used to measure the crystallinities of the hydrogel samples in their swollen state. In a typical DSC measurement, the hydrogel sample was sealed in a hermetic Tzero pan and a reference Tzero pan without any sample was also contained. The sample was heated from 50 °C to 250 °C at 10 °C·min^−1^ under a nitrogen flow of 50 mL·min^−1^. A narrow peak appeared in the DSC heating curve ranging from 200 °C to 250 °C, corresponding to melting of the PVA crystalline domains. The enthalpy for the melting of the crystalline domains per unit mass of the sample (*H_crystalline_*) can be calculated by the integration of the endothermic transition from 200 to 250 °C. Therefore, the crystallinity in the swollen sample (*X_swollen_*) can be calculated as *X_swollen_* = *H_crystalline_*/*H°_crystalline_*, where *H°_crystalline_* = 138.6 J/g was the enthalpy of the fusion of 100 wt% crystalline PVA measured at the equilibrium melting point (T^0^_m_). To rule out the effect of water content, the normalized crystallinity was calculated as *X_normalization_* = *X_swollen_*/w_s_, where w_s_ was the solid content of the hydrogel.

### 4.5 Measurement of Water Content

The hydrogel samples were dried in an oven at 60 °C for 3 days till totally dried to measure the water content. The weight of the sample before and after drying were measured as W_swollen_ and W_dry_, and water content (W) can be obtained from the following equation: W  =  (1 ‐ W_dry_/W_swollen_)  × 100%.

### 4.6 Mechanical Tests

All mechanical tests of the hydrogels were conducted on a universal tensile machine (Instron Model 5567) with a 5 kN loading cell under room temperature. The cubic hydrogel block (40 mm × 40 mm × 40 mm) was sliced into thin sheets (approximately 1 mm thickness) along XY, XZ and YZ planes, which were subsequently cut into dumbbell‐shaped specimens with a gauge section of 17 mm length and 4 mm width for mechanical tests along the X, Y, and Z directions. The thickness of the specimens was measured by a vernier caliper and was typically around 1 mm. For the tensile tests and tensile loading‐unloading tests, the speed was set at 50 mm/min. For the compression test, the cylindrical hydrogel specimens with a height of 10 mm and a diameter of 8 mm were used, and the compression speed was set to 3 mm/min. The strain of the hydrogel specimen was defined as the ratio of the length change to the initial length, while the stress was calculated by dividing the force by the initial cross‐sectional area of the specimen. The elastic modulus of the hydrogel specimens was calculated by fitting the slope of the linear region in the initial stage of the stress‐strain curve. The toughness of the hydrogel specimens was calculated by the area under the stress‐strain curve. The fracture energy was measured by the pure shear testing using the rectangular specimens with a height of 30 mm, a width of 40 mm and a thickness of approximately 1 mm. The initial notch length of the notched specimens was 20 mm. The initial clamp distance was 10 mm and the tensile rate was 50 mm/min. Briefly, a force‐displacement curve was measured for both the notched and unnotched sample with the same initial dimensions under the same testing condition. With denoting of the sample cross section area as A, the critical distance where the notch turned into a running crack as L_c_, and the work done to an unnotched sample to reach L_c_ as U(L_c_), the fracture energy can be calculated as Γ  =  *U*(*L*_c_)/*A*, where $U\;\left(L_{c}\right)=\int_{0}^{L_{c}}F\;\left(L\right)\;dL$ and F was the applied force. For the loading‐unloading tests, the dissipated energy and dissipation coefficient were calculated as the area of the hysteresis loop and the area of the hysteresis loop divided by the area under the loading curve, respectively.

### 4.7 Fatigue Tests

Fatigue tests were performed on a universal tensile machine (Univert S2, CellScale) with 200 N loading cell in 1 M sodium phytate solution. The single‐notch method was used to measure the fatigue threshold for evaluation of fatigue resistance. Cyclic loading with an applied strain (λ_a_) was performed on both notched and unnotched hydrogel samples with identical dimensions. For the notched specimen, the initial pre‐crack length was less than one‐fifth of its width, and a digital camera was employed to record crack extension per cycle (dc/dN). The energy release rate (G) was calculated according to G = 2kcW, where k was determined to be 3/(λ_a_ + 1)^0.5^, c was the crack length, and W was the strain energy density of the unnotched specimen applied with the same λ_a_. W was calculated by integrating the loading curve when the loading‐unloading curves became stable. By varying λ_a_, we acquired the plot of dc/dN versus the applied G. The fatigue threshold was obtained by linearly extrapolating the curve of dc/dN versus G to the intercept with the abscissa.

### 4.8 Quasi‐Static Puncture Tests

The quasi‐static puncture tests were conducted following the standard ASTM F3007, using the hydrogel samples with a length of 40 mm, a width of 40 mm and a thickness of 1 mm. The hydrogel samples were secured between two steel hoop, and quasi‐static puncture tests were performed on a universal testing machine (Instron Model 5567). A spherical‐tipped loading nose (stainless steel, radius of 4 mm) was punctured onto the center of the samples at a quasi‐static speed of 10 µm/s until perforation. The puncture stress (σ) was calculated by dividing the puncture force by the hydrogel sample thickness (T), i.e., $\sigma =\frac{F}{T}$, and the maximum puncture stress was defined as the puncture strength. The puncture energy was computed as the area under the puncture stress versus displacement curve, i.e., $E=\int_{0}^{D}\;\sigma \;\left(D\right)\;dD$, where D was the displacement.

### 4.9 Impact Resistance Tests

The drop‐weight impact test was performed to demonstrate the impact resistance of hydrogels, where a 400 g stainless‐steel impactor (spherical tip radius: 4 mm) was dropped from a height of 1 m (i.e., nominal impact energy of 3.92 J) onto the hydrogel sheets to determine whether it would penetrate the hydrogel sheet and shatter the underlying glass slide. These hydrogel sheets with length of 40 mm, width of 40 mm and thickness of 2 mm were fixed between two steel hoops.

## Author Contributions

**Bolin Wan**: methodology, investigation, formal analysis, writing – original draft, data curation. **Xule Yang**: methodology, data curation. **Liju Xu**: conceptualization, methodology, data curation, funding acquisition, writing – original draft, writing – review and editing. **Dong Qiu**: conceptualization, supervision, funding acquisition, writing – review and editing.

## Funding

National Natural Science Foundation of China (grant 52225309 and 52473284) and the Beijing Natural Science Foundation (grant L234014 and JQ24008).

## Conflicts of Interest

The authors declare no conflicts of interest.

## Supporting information




**Supporting File 1**: advs77852‐sup‐0001‐SuppMat.docx.

**Supporting File 2**: advs77852‐sup‐0002‐MovieS1.mp4.

**Supporting File 3**: advs77852‐sup‐0003‐MovieS2.mp4.

## Data Availability

The data that support the findings of this study are available from the corresponding author upon reasonable request.
