# Supplementary material for: Strengthening Constitutive Motifs in Isotropic Fibrous Hydrogel for Exceptional Damage Resistance
Source: Adv Sci (Weinh). 2026 Sep 27:e77852. Online ahead of print. doi: 10.1002/advs.77852 (PMC13616275; doi:10.1002/advs.77852)
Supplement: Supplementary file 1 [file ADVS-9999-e77852-s002.docx]

Supporting Information

**Strengthening constitutive motifs in isotropic fibrous hydrogel for exceptional damage resistance**

*Bolin Wan, Xule Yang, Liju Xu*, and Dong Qiu**

**Supplementary movies**

**Movie S1. Drop-weight impact test of hydrogels.** This movie showed that the DCIF-PVA, IF-PVA and IM-PVA hydrogel sheets were impacted by a 400 g steel impactor (spherical tip radius of 4 mm) from the same height of 1 m to determine if the impactor would penetrate the hydrogel sheets and shatter the underlying glass slides. The IF-PVA and IM-PVA hydrogels ruptured easily upon impact and failed to protect the underlying glass slides form breaking. In stark contrast, the DCIF-PVA hydrogel effectively resisted the impact and kept the underlying glass slide intact, revealing its excellent damage resistance. The movie was shown at real-time speed at 30 frames per second.

**Movie S2. This movie was shot at the same time with movie S1 at a close range.**

**Materials**

***Materials.*** Polyvinyl alcohol (PVA, M.W. 89000-98000, 99% hydrolyzed, Aldrich), N-Methylpyrrolidone (NMP, A.R. grade, Innochem), ethanol (A.R. grade, Concord Technology), phytic acid solution (PA, Aldrich), sodium hydroxide (Sinopharm Chemical Reagent Co. Ltd.) and sodium citrate (CA, Innochem) were purchased and used without further purification. Deionized water (18.2 MΩ·cm) was used and generated by a Genie PURIST system.

**Supplementary figures and tables**

**Figure S1. Preparation of the DCIF-PVA, IF-PVA and IM-PVA hydrogels from the same PVA/NMP gel.** Since all three hydrogels shared identical chemical compositions, it enabled direct structural comparison to demonstrate the effect of network structure on their mechanical properties.

**Figure S2.** **Comparison of the** **fracture energy of the** **DCIF-PVA, IF-PVA and IM-PVA hydrogels.** The stress-strain curves of the unnotched and notched (A) DCIF-PVA, (B) IF-PVA and (C) IM-PVA hydrogels. In the pure shear testing, the DCIF-PVA hydrogel exhibited the highest critical strain (ε_c_) and strength (σ_c_) among the three hydrogels, thus achieving the highest fracture energy.

**Figure S3.** **Water contents of the DCIF-PVA, IF-PVA, and IM-PVA hydrogels.** The error bars represented the standard deviation; sample size n = 3.

**Figure S4.** **Comparison of the mechanical properties of the DCIF-PVA, IF-PVA, and IM-PVA hydrogels with the nearly identical water content.** (A) Tensile stress-strain curves and (B) the corresponding modulus and strength of the DCIF-PVA, IF-PVA, and IM-PVA hydrogels with the nearly identical water content of ~40%.

**Figure S5. Comparison of the mechanical properties of the DCIF-PVA and Sal-IM-PVA hydrogels.** (A) Representative tensile stress-strain curves, (B) tensile modulus and strength, and (C) the water content of the DCIF-PVA and Sal-IM-PVA hydrogels. The error bars represented standard deviations; sample size n = 3. The Sal-IM-PVA hydrogel fabricated by salting out the conventional IM-PVA hydrogel showed much lower modulus and strength even at a lower water content than the developed DCIF-PVA hydrogel, highlighting the key role of the DCIF network for enhanced mechanical properties.

**Figure S6. Comparison of the fracture energy of the DCIF-PVA and Sal-IM-PVA hydrogels.** The stress-strain curves of the unnotched and notched (A) Sal-IM-PVA hydrogel. (B) The fracture energy of the DCIF-PVA and Sal-IM-PVA hydrogels. The DCIF-PVA hydrogel exhibited superior fracture energy than the Sal-IM-PVA hydrogel. The error bars represented standard deviations; sample size n = 3.

**Figure S7.** **Comparison of the crystallinity of the DCIF-PVA and Sal-IM-PVA hydrogels.** (A) DSC thermographs and (B) the corresponding crystallinities of DCIF-PVA and Sal-IM-PVA hydrogels.

**Figure S8. Comparison of mechanical properties of the DCIF-PVA hydrogel in the X, Y, and Z directions.** (A) Tensile modulus (22.10 ± 1.52 MPa, 22.62 ± 0.43 MPa, 20.64 ± 2.06 MPa), (B) tensile strength (20.15 ± 1.96 MPa, 19.32 ± 1.30 MPa, 20.43 ± 1.35MPa), (C) toughness (126.48 ± 5.30 MJ/m^3^, 122.50 ± 7.78 MJ/m^3^, 134.00 ± 1.41 MJ/m^3^), and (D) fracture energy (242.01 ± 2.03 kJ/m^2^, 244.36 ± 6.03 kJ/m^2^, 251.10 ± 5.67 kJ/m^2^) of the DCIF‑PVA hydrogel along the X, Y, and Z directions. For each mechanical property, no significant difference was observed among the X, Y, and Z directions (one‑way ANOVA, p > 0.05), supporting the mechanical isotropy. Accordingly, the values obtained from the X direction were taken as the representative results. The error bars represented the standard deviation; sample size n = 3.

**Figure S9. Compressive performance of the DCIF-PVA, IF-PVA, and IM-PVA hydrogels.** (A) Compressive stress-strain curves and (B) the modulus and toughness of the DCIF-PVA, IF-PVA, and IM-PVA hydrogels. The error bars represented the standard deviation; sample size n = 3.

**Figure S10. Cyclic tensile properties of the DCIF-PVA, IF-PVA, and IM-PVA hydrogels.** Cyclic tensile loading-unloading stress-strain curves of the (A) DCIF-PVA hydrogel under varied strains (λ = 1, 3, 5, 7, 9), (B) IF-PVA hydrogel under varied strains (λ = 1, 3, 5, 7), (C) IM-PVA hydrogel under varied strains (λ = 1, 3), and (D) the corresponding dissipated energy and dissipation coefficient. The crosses in D indicated the hydrogel fracture at the applied strains. The error bars represented the standard deviation; sample size n = 3.

**Figure S11. Fatigue resistance behavior of the DCIF-PVA hydrogel.** Validation of the fatigue threshold of 5.54 kJ/m^2^ for the DCIF-PVA hydrogel by the single-notch test. Photographs were taken at the 1st, 1000th and 5000th cycle. Scale bar: 2 mm.

**Figure S12. The azimuthal integration curves of the DCIF‑PVA hydrogel at strains of 0 and 6.** These curves were derived from 2D SAXS patterns from 0 to 180° at a fixed *q*-range of 0.06 ± 0.01 Å^-1^.

**Figure S13.** **Comparison of the mechanical properties of the DCIF-PVA-CA and Sal-IM-PVA-CA hydrogels.** (A) Tensile stress-strain curves and (B) tensile modulus and strength of the DCIF-PVA-CA and Sal-IM-PVA-CA hydrogels. The error bars represented standard deviations; sample size n = 3.

**Figure S14.** **Comparison of the fracture energy of the DCIF-PVA-CA and Sal-IM-PVA-CA hydrogels.** The stress-strain curves of the unnotched and notched (A) DCIF-PVA-CA and (B) Sal-IM-PVA-CA hydrogels. (C) The fracture energy of the DCIF-PVA-CA and Sal-IM-PVA-CA hydrogels. The error bars represented standard deviations; sample size n = 3.

**Figure S15. Comparison of the mechanical performances of the DCIF-PVA-annealing and IM-PVA-annealing hydrogels.** (A) Tensile stress-strain curves and (B) tensile modulus and strength of the DCIF-PVA-annealing and IM-PVA-annealing hydrogels. The error bars represented standard deviations; sample size n = 3.

**Figure S16. Comparison of the fracture energy of the DCIF-PVA-annealing and IM-PVA-annealing hydrogels.** The stress-strain curves of the unnotched and notched (A) DCIF-PVA-annealing and (B) IM-PVA-annealing hydrogels. (C) The fracture energy of the DCIF-PVA-annealing and IM-PVA-annealing hydrogels. The error bars represented standard deviations; sample size n = 3.

**Figure S17.** **Comparison of the** **puncture resistance of the DCIF-PVA, IF-PVA and IM-PVA hydrogels.** (A) Puncture strength and (B) puncture energy of DCIF-PVA, IF-PVA and IM-PVA hydrogels. The error bars represented standard deviations; sample size n = 3.

**Table S1. Mechanical property comparison of DCIF-PVA hydrogels with reported isotropic and anisotropic hydrogels, including strength, toughness and fracture energy**

|  | Sample | Strength  (MPa) | Toughness (MJ/m^3^) | Fracture energy (kJ/m^2^) | Ref. |
| --- | --- | --- | --- | --- | --- |
| Isotropic hydrogels | DCIF-PVA | 20.2 | 126.5 | 242.0 | This work |
|  | HC-SI | 7.4 | 40.0 | N/A | *Matter* 4 (2021): 1919-1934. |
|  | PAAM | 1.2 | 87.7 | 26.2 | *Nat. Mater.* 23 (2024): 131-138. |
|  | PCP | 1.9 | 68.0 | 43.1 | *Angew. Chem. Int. Ed.* 63 (2024): e202316375. |
|  | PEG | 5.5 | 22.0 | 3.6 | *Science* 372 (2021): 1078-1081. |
|  | PNA | 9.1 | 33.7 | 171.1 | *Adv. Funct. Mater.* 33 (2023): 2210395. |
|  | Alg/PVA | 15.0 | N/A | 39.0 | *Adv. Funct. Mater.* 32 (2022): 2204823. |
|  | SAPAM | 0.4 | N/A | 78.5 | *Adv. Mater.* 37 (2025): 2417795. |
|  | PVA-PA | 25.0 | N/A | 120.0 | *Adv. Mater*. 35 (2023): 2209913. |
|  | PVA/GO | 8.8 | N/A | 85.7 | *Adv. Mater.* 35 (2023): 2207587. |
|  | SEWA | 11.2 | 82.3 | 25.4 | *Adv. Mater.* 35 (2023): 2210624. |
|  | HA-PEG | 15.3 | 138.0 | 6.4 | *Nat. Mater.* 22 (2023): 1253-1260. |
| Anisotropic hydrogels | AFH | 14.0 //  1.0 ⊥ | 154.0∥  3.0 ⊥ | 153.0 // | *Nat. Commun.* 15 (2024): 118. |
|  | HA-PVA | 23.5 //  3.0 ⊥ | 210.0 //  15.0 ⊥ | 170.0 // | *Nature*  590 (2021): 594-599. |
|  | Pre-PVA | 11.0 //  4.0 ⊥ | 40.0 //  20.0 ⊥ | N/A | *Chin. Chem. Lett.* 33 (2022): 871-876. |
|  | PF | 8.0 //  1.5 ⊥ | 72.0 //  10.0 ⊥ | N/A | *Mater. Horiz.* 11 (2024): 2131-2142. |
|  | FC-EtFe | 6.5 //  1.8 ⊥ | 70.0 //  6.5 ⊥ | N/A | *Adv. Funct. Mater.* 32 (2022): 2203610. |
|  | PAAc/PAAm | 20.0 //  10.0 ⊥ | 45.0 //  16.0 ⊥ | N/A | *Small* 12 (2016): 4386-4392. |
|  | FC-A PVA | 3.0 //  0.3 ⊥ | 8.0 //  0.4 ⊥ | 116.0 // | *Adv. Mater.* 33 (2021): 2102011. |
|  | ACH | 8.0 //  0.6 ⊥ | N/A | 2.0 // | *Chem. Mater*. 30 (2018): 5175-5183. |

N/A indicated the corresponding data were not available from the references.
